# Effect of protective ventilation on organ-specific cytokine production in an experimental postoperative sepsis model

**DOI:** 10.1186/cc12050

**Published:** 2013-03-19

**Authors:** J Sperber, M Lipcsey, A Larsson, A Larsson, J Sjölin, M Castegren

**Affiliations:** 1 Centre for Clinical Research Sörmland, Uppsala University, Uppsala, Sweden; 2 Surgical Sciences, Uppsala University, Uppsala, Sweden; 3 Medical Sciences, Uppsala University, Uppsala, Sweden

## Introduction

Low tidal volume (VT) ventilation in intensive care patients without lung injury attenuates the systemic inflammatory response [1]. The contribution of the specific organ inflammatory responses to the systemic picture remains to be elucidated. We investigated the effect of low VT ventilation compared with medium high VT on hepatic, splanchnic and cerebral cytokine responses in an experimental large animal postoperative sepsis model.

## Methods

Twenty pigs, group Protective Ventilation (PV), were ventilated with low VT (6 ml/kg) and PEEP 10 cmH_2_O while 10 pigs, group Control (C), were ventilated with a VT of 10 ml/kg and PEEP 5 cmH_2_O. Catheters were introduced into an artery, the jugular bulb, the hepatic vein and the portal vein. Laparotomy for 2 hours simulated a surgical procedure after which baseline ensued and a continuous endotoxin infusion was started at 0.25 μg/kg/hour for 5 hours. Differences were analyzed with ANOVA for repeated measures.

## Results

TNFα levels were higher in the hepatic vein than in the artery, the jugular bulb and the portal vein. IL-6 levels were higher in the artery and the jugular bulb compared with the portal and hepatic veins. IL-10 levels were higher in the portal vein compared with the jugular bulb and hepatic vein. The organ-specific IL-10 concentrations were all higher than the arterial concentration. Comparison between the ventilation groups showed that TNFα, IL-6 and IL-10 in the hepatic vein were higher in group C compared with group PV at the end of the experiment. Peak concentrations of TNFα and IL-6 in the portal vein were higher in group C compared with group PV.

## Conclusion

In this experiment TNFα was mainly generated in the liver while the results point to significant nonhepatic IL-6 and IL-10 production. Ventilation with low VT and medium-high PEEP attenuated hepatic and splanchnic cytokine production compared with medium-high VT and lower PEEP.
